# Inhibitory effects of prepulse stimuli on the electrophysiological responses to startle stimuli in the deep layers of the superior colliculus

**DOI:** 10.3389/fnins.2024.1446929

**Published:** 2024-08-14

**Authors:** Yu Ding, Huan Jiang, Na Xu, Liang Li

**Affiliations:** ^1^School of Psychology, Beijing Language and Culture University, Beijing, China; ^2^School of Psychological and Cognitive Sciences, Peking University, Beijing, China; ^3^Division of Brain Sciences, Changping Laboratory, Beijing, China

**Keywords:** prepulse inhibition, superior colliculus, inferior colliculus, frequency following response, tinnitus

## Abstract

**Background:**

Prepulse inhibition (PPI) is a phenomenon where a weak prepulse stimulus inhibits the startle reflex to a subsequent stronger stimulus, which can be induced by various sensory stimulus modalities such as visual, tactile, and auditory stimuli.

**Methods:**

This study investigates the neural mechanisms underlying auditory PPI by focusing on the deep layers of the superior colliculus (deepSC) and the inferior colliculus (IC) in rats. Nineteen male Sprague-Dawley rats were implanted with electrodes in the left deepSC and the right IC, and electrophysiological recordings were conducted under anesthesia to observe the frequency following responses (FFRs) to startle stimuli with and without prepulse stimuli.

**Results:**

Our results showed that in the deepSC, narrowband noise as a prepulse stimulus significantly inhibited the envelope component of the startle response, while the fine structure component remained unaffected. However, this inhibitory effect was not observed in the IC or when the prepulse stimulus was a gap.

**Conclusion:**

These findings suggest that the deepSC plays a crucial role in the neural circuitry of PPI, particularly in the modulation of the envelope component of the startle response. The differential effects of narrowband noise and gap as prepulse stimuli also indicate distinct neural pathways for sound-induced PPI and Gap-PPI. Understanding these mechanisms could provide insights into sensory processing and potential therapeutic targets for disorders involving impaired PPI, such as tinnitus.

## 1 Introduction

Prepulse inhibition (PPI) is a phenomenon in which a prepulse stimulus inhibits the startle reflex. PPI can be induced by various sensory stimulus modalities, such as visual, tactile, and auditory stimuli, and is widely used to study sensorimotor gating and central nervous system functions (Graham, 1975). Specifically, auditory PPI can be induced by pure tones, narrowband noise, and gaps in background noise as prepulse stimuli, making it an important tool for studying various neuropsychiatric disorders, such as schizophrenia, attention deficit hyperactivity disorder, and tinnitus (Ashare et al., 2007; Lei et al., 2014). PPI is a sensorimotor gating mechanism common to both humans and animals. According to the “processing-protection” theory, the appearance of a weak sensory stimulus triggers an “immediate detection response” and automatically initiates a gating process to attenuate the processing of subsequent strong interfering stimuli until the perceptual processing of the initial stimulus is completed (Graham, 1975). Research has confirmed that the magnitude of PPI is primarily regulated by the Prepulse stimulus, reflecting the brain’s processing of information from the Prepulse stimulus (Swerdlow et al., 2001). The deep layers of the superior colliculus (deepSC) are key nodes in the PPI circuit, integrating signals from different sensory channels and regulating the startle reflex (Zou et al., 2007; Li et al., 2008, 2009). The inferior colliculus (IC) is a crucial nucleus in the auditory pathway, transmitting signals to the superior colliculus and higher central nervous system structures (Cadusseau and Roger, 1985; Chandrasekaran and Kraus, 2010). Additionally, the IC plays an integral role in the descending auditory pathway, contributing to the feedback and modulation of auditory information processing (reviewed in Malmierca, 2004).

Normal PPI has been observed in decorticated rats (Li and Frost, 2000), infants (Huggenberger et al., 2011), and sleeping human subjects (Horner et al., 1997), indicating that PPI is an automatic process. The underlying neural circuits are located in the subcortical brainstem, including the auditory midbrain (inferior colliculus), the deep and intermediate layers of the superior colliculus, and the pedunculopontine tegmental nucleus (PPTg). The inferior colliculus has extensive neural projections to the superior colliculus, which then projects to the PPTg. Additionally, the limbic system, motor system, and sensory cortex have extensive projections to the PPI circuit, allowing PPI to be modulated by various factors. These connections provide the anatomical basis for sensorimotor gating being regulated by both bottom-up and top-down factors.

Fendt et al. found that lesions in the superior colliculus decreased PPI by only about 45% (Fendt et al., 1994, 2001), while another study reported that lesions in the inferior colliculus completely disrupted PPI (Leitner and Cohen, 1985). This suggests that the basic PPI circuit may have multiple branches: a fast pathway bypassing the superior colliculus and a slower pathway passing through the superior colliculus. This multi-branch neural circuit facilitates the modulation of PPI by attention. For example, the IC-VNTB (ventral nucleus of the trapezoid body)-CRNs (cochlear root neurons) pathway mediates rapid PPI over short intervals (Barioni et al., 2024), while the IC-PPTg-PnC (pontine reticular nucleus) pathway also bypasses the deepSC (Yeomans et al., 2006). The sequential circuit connecting the IC, deepSC, PPTg, and PnC may constitute the slower midbrain PPI pathway (see review by Lei et al., 2021). Additionally, some brain regions, such as the lateral globus pallidus (LGP), are also involved in the neural transmission of PPI (Moreno-Paublete et al., 2017).

Numerous studies have found that a gap embedded in continuous background noise can also serve as a prepulse stimulus to inhibit the startle reflex, known as Gap-PPI (Ison et al., 1998; Huang et al., 2007; Zou et al., 2007; Li et al., 2008; Sun et al., 2014). Gap-PPI is commonly used in tinnitus research. Theoretically, tinnitus patients continuously hear a background sound, so the energy gap embedded in real background noise may be filled by the tinnitus sound, reducing or eliminating Gap-PPI. Researchers often use Gap-PPI to establish tinnitus models in experimental rats, taking advantage of PPI’s ability to be measured without requiring active reporting from subjects (Turner et al., 2006; Yang et al., 2007).

While previous studies have primarily focused on recording the neural expression of the Prepulse stimulus, this study emphasizes recording the neural characteristics of the startle stimulus in the midbrain. To investigate the neural processing characteristics of startle stimuli and their Prepulse inhibition in the deepSC and IC, this study employs frequency following responses (FFRs) as the recording method. FFRs are neural phase-locking responses that reflect the temporal and spectral characteristics of the auditory system’s processing of sound stimuli, widely used in auditory research (Marsh and Worden, 1969; Weinberger and Oleinick, 1970; Du et al., 2009a; Chandrasekaran and Kraus, 2010). Previous studies have successfully recorded FFRs in the IC of rats, and FFRs recorded from human scalp electrodes are also believed to originate from the auditory midbrain (Smith et al., 1975; Sohmer et al., 1977; Chandrasekaran and Kraus, 2010).

In this study, electrodes were implanted in the deepSC and IC of rats to record and analyze the electrophysiological responses of these two brain regions to different types of prepulse stimuli (such as narrowband noise or energy gaps) on the startle stimulus. The study aims to address the following two scientific questions: 1) whether the fine structure or envelope components of startle stimuli in the deepSC and IC are affected by prepulse stimuli, and 2) if prepulse stimuli affect the expression of startle stimuli, which specific neural components are altered. By comparing the electrophysiological data from these two brain regions, we aim to elucidate the distinct roles of the deepSC and IC in PPI and gap-PPI modulation and provide new insights into the neural basis of PPI.

## 2 Materials and methods

### 2.1 Experimental animals

Nineteen group-housed male Sprague-Dawley rats (12 weeks old, weighing 270–350 g, purchased from Beijing Vital River Laboratory Animal Technology Co., Ltd.) were implanted with electrodes in the left deepSC and the right IC. Implanting electrodes in the deepSC and IC on opposite sides can avoid interference from the stereotaxic arm, thereby improving the success rate of the surgery. It is important to emphasize that there are significant gender differences in PPI, and in female animals, PPI may be regulated by factors such as the menstrual cycle and hormonal changes (Lehmann et al., 1999; Wu et al., 2019). Therefore, this study used only male rats. After purchase, the rats were housed in pairs in each cage. During the experiment, the rats were provided with sufficient food and water. The animal housing room maintained an average temperature of 24 ± 2°C and a 12-h light/dark cycle. The handling of animals in this study complied with the guidelines of the Beijing Experimental Animal Center and the Policy on the Use of Animals and Humans in Neuroscience Research approved by the Society for Neuroscience (2006). All experimental procedures were approved by the Human and Animal Experiment Ethics Committee of the School of Psychological and Cognitive Sciences, Peking University (approval of IRB Protocol 20190302e), and every effort was made to minimize animal suffering.

### 2.2 Surgery and electrode implantation

Before surgery, each rat was weighed and anesthetized with an intraperitoneal injection of chloral hydrate solution (10% concentration, dissolved in saline) at a dose of 500 mg/kg. The anesthesia status was assessed by testing the toe pinch reflex; anesthesia was deemed effective when the rats lost pain response. Throughout the experiment, the anesthesia status of the rats was monitored via the toe pinch reflex, and additional doses of anesthesia (10 mg per injection) were administered if the rats showed signs of waking. The rats were fasted for 8 hours before surgery to prevent abnormal food accumulation after anesthesia. After anesthesia, an electric heating pad was used to maintain the rats’ body temperature at 37 degrees Celsius, and the position of the rats’ mouths was adjusted to prevent the tongue from obstructing breathing.

The rats were fixed in a small animal stereotaxic apparatus (KOPF 902, WPI, USA) with the head stabilized using a nose clamp and hollow metal ear bars (without damaging the tympanic membrane). The head was adjusted to maintain a horizontal position. The fur on the top of the head was shaved, and a midline incision was made on the scalp to expose the skull. The bregma and lambda were marked, and the stereotaxic apparatus was adjusted to align the bregma and lambda at the same horizontal level. Saline was used to keep the surgical field clear, and proper disinfection was performed throughout the surgery. A screw was inserted into the skull over the frontal and parietal cortex to serve as a reference and ground electrode. The target brain regions were marked on the skull based on the rat brain atlas coordinates, and small holes (approximately 0.75 mm in diameter) were drilled using a dental drill.

Homemade stainless steel electrodes (insulated with silicone tubing, with only the 0.25 mm tip exposed, resistance 10–25 kΩ) were vertically inserted into the left deepSC and the right IC central nucleus. The electrodes were fixed in place with dental cement. According to the rat brain atlas based on the stereotaxic coordinates of Paxinos and Watson (1996), the coordinates for the deepSC relative to the bregma were AP: −6.6 mm, ML: ± 1.5 mm, DV: −4.5 to −5.0 mm, and for the IC central nucleus were AP: −8.8 mm, ML: ± 1.5 mm, DV: −4.5 to −5.0 mm. After surgery, the rats were transferred to the electrophysiological recording room. Once anesthesia and vital signs were stable, data collection began immediately.

### 2.3 Experimental stimuli

The sound stimuli used in the experiment were generated using MATLAB (the MathWorks, Natick, MA). Narrowband noise was generated by creating a Gaussian white noise segment at a sampling rate of 24414 Hz and 16-bit sampling accuracy, then passing it through a 512-point bandpass filter to obtain the corresponding center frequency and bandwidth of the narrowband noise.

In this experiment, the Prepulse stimuli included a 2100 Hz narrowband noise (1/3 octave, 150 ms duration, 60 dB SPL) and a 50 ms energy gap embedded in background noise (2100 Hz narrowband noise, 1/3 octave, 900 ms duration, 60 dB SPL). These Prepulse stimuli simulated the PPI and Gap-PPI conditions from previous behavioral experiments (Du et al., 2010; Ding et al., 2019; Chen et al., 2021). To prevent spectral leakage, both the Prepulse stimuli and the background noise had 5 ms Hanning window rise and fall times. The startle stimulus was a 3000 Hz narrowband noise (1/3 octave, 100 ms duration, 90 dB SPL). Due to the ineffectiveness of longer rise times in inducing the startle reflex, the startle stimulus had only a 1 ms Hanning window rise time, with the same fall time as the Prepulse stimuli. The interstimulus interval (ISI) between the Prepulse and startle stimuli was 50 ms. Narrowband noise was used to effectively record frequency following responses.

### 2.4 Experimental equipment

Electrophysiological recordings were performed using a TDT system. Bilateral startle and prepulse stimuli were delivered through ear tubes. Sound stimuli were generated by the TDT (Tucker-Davis Technologies, FL, USA) system’s signal processing station (RZ6 model), converted to digital signals, filtered, and output through TDT system MF1 speakers. Insulated PVC ear tubes (10 cm) connected the speakers to the rats’ external ear canals, and sound pressure levels were calibrated using a sound level meter (Larson Davis Audiometer Calibration and Electroacoustic Testing System, AUDit and System 824) positioned at the rat’s head (Xu et al., 2019).

All electrophysiological recordings were conducted in a custom-made electromagnetic shielded box lined with sound-absorbing foam. The rats’ electrophysiological signals were received by a front-end amplifier (TDT RA16P Medusa), amplified by 80 dB, and transmitted to the TDT signal processing station for online filtering (5 – 5000 Hz) and 50 Hz notch filtering. Signals were averaged across repeated trials and transmitted to a computer. The recording time for each stimulus condition was 1000 ms, and raw data were sampled at 24414 Hz using the TDT system’s BioSig software.

### 2.5 Experimental procedure

The experiment consisted of four conditions: (1) startle stimulus only, with the startle stimulus presented at 520 ms after trial onset; (2) Prepulse and startle stimuli, with the Prepulse stimulus presented at 320 ms (150 ms duration) and the startle stimulus at 520 ms; (3) startle stimulus in background noise, with background noise presented from 20 ms to 920 ms and the startle stimulus at 520 ms; (4) energy gap in background noise and startle stimulus, with background noise presented from 20 ms to 920 ms, a 50 ms energy gap at 420 ms, and the startle stimulus at 520 ms. All conditions were binaurally delivered, with each condition repeated 50 times. Electrophysiological signals were averaged across trials, and the interval between startle stimuli was 1 s. Conditions were balanced across subjects.

### 2.6 Data analysis

Using MATLAB, frequency following response segments of 100 ms were extracted starting from the onset of the startle stimulus for analysis. Theoretically, the fine structure energy of a steady-state narrowband noise with a center frequency of c and bandwidth of b is distributed around c, while the envelope component energy is distributed within the 0-b range (Longtin et al., 2008). Therefore, the responses were band-pass filtered at 1000 Hz to isolate the low-frequency envelope and high-frequency fine structure components. Fast Fourier Transform (FFT) was performed on the purified fine structure and envelope components to obtain amplitude values (Ampf) for each frequency.

The study focused on changes in response amplitude and relative amplitude of the envelope and fine structure components in the presence or absence of Prepulse stimuli. The envelope and fine structure amplitudes were normalized to the response without Prepulse stimuli, producing a ratio used for further analysis. Relative amplitude was inherently normalized, eliminating individual differences.

The envelope component amplitude was defined as:


$$F\,F\,R\,E\,n\,v=\sum_{3}^{b}A\,m\,p\,f$$

The fine structure amplitude was defined as:


$$F\,F\,R\,T\,F\,S=\sum_{f\,l\,c}^{f\,h\,c}A\,m\,p\,f$$

The relative envelope component amplitude was defined as:


$$F\,F\,R\,E\,n\,v\,\_\,r\,e\,l\,a\,t\,i\,v\,e=\sum_{3}^{b}A\,m\,p\,f/\sum_{3}^{5000}A\,m\,p\,f$$

The relative fine structure amplitude was defined as:


$$F\,F\,R\,T\,F\,S\,\_\,r\,e\,l\,a\,t\,i\,v\,e=\sum_{f\,l\,c}^{f\,h\,c}A\,m\,p\,f/\sum_{3}^{5000}A\,m\,p\,f$$

The data were analyzed using SPSS 20.0 software (SPSS Inc., Chicago, Illinois) with repeated measures ANOVA, α = 0.05.

### 2.7 Histological examination

After all recordings were completed, the rats were euthanized with an overdose of chloral hydrate at a dose of 1500 mg/kg. Electrode positions were marked using a direct current lesion method. Positive direct current was applied to the recording brain regions (500 μA, 10 s), with the cathode attached to the moistened scalp. After brain extraction, the tissue was preserved in a fixative solution containing 10% formalin and 30% sucrose. Two weeks later, the brain tissue was fixed and sectioned using a cryostat (Leica CM1950, Germany) at −20°C, producing 40 μm coronal sections. The lesion sites on the sections were located by referring to a brain atlas (Paxinos and Watson, 1996).

## 3 Results

### 3.1 Histological examination results

**FIGURE 1:**
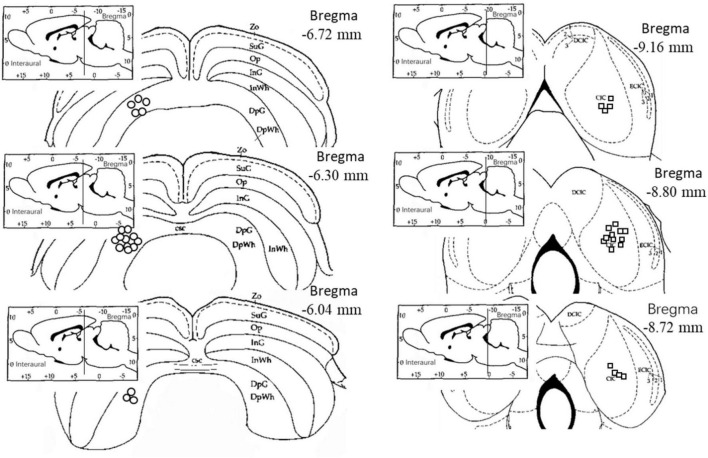
Electrode location diagram. The circles indicate the electrodes in the left deep layers of the superior colliculus, while the squares indicate the electrodes in the right central nucleus of the inferior colliculus. The electrode locations were observed and recorded on the rat brain atlas using frozen sections (Paxinos and Watson, 1996). Zo, Zonular Layer of the Superior Colliculus; SuG, Superficial Gray Layer of the Superior Colliculus; Op, Optic Layer of the Superior Colliculus; InG, Intermediate Gray Layer of the Superior Colliculus; InWh, Intermediate White Layer of the Superior Colliculus; DpG, Deep Gray Layer of the Superior Colliculus; DpWh, Deep White Layer of the Superior Colliculus; csc, Commissure of the Superior Colliculus; CIC, Central Nucleus of the Inferior Colliculus; DCIC, Dorsal Cortex of the Inferior Colliculus; ECIC, External Cortex of the Inferior Colliculus.

Histological examination revealed that 19 electrodes were accurately positioned in the right central nucleus of the inferior colliculus (CIC) and 19 electrodes were accurately positioned in the left deep gray layer of the superior colliculus (DpG). The electrophysiological data obtained from the correctly positioned electrodes were included in the subsequent analysis and statistical tests (Figure 1).

### 3.2 Responses to startle stimuli in the deepSC and IC

**FIGURE 2:**
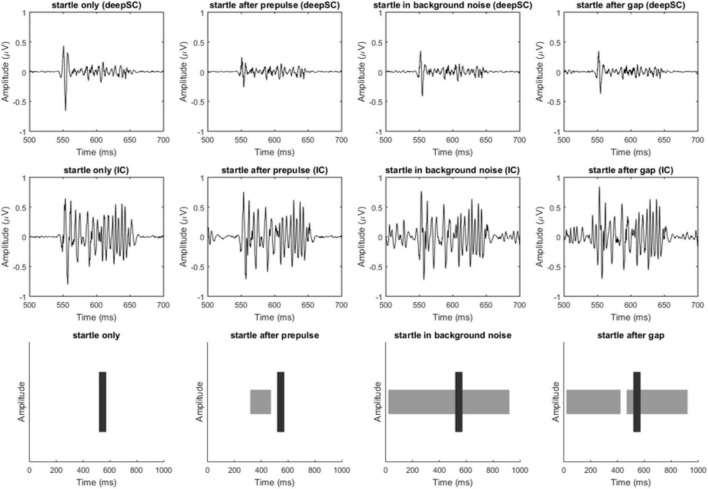
Filtered response diagrams of the deepSC and IC, and stimulus diagram. The first row shows the response diagrams of the deepSC, the second row shows the response diagrams of the IC, and the third row illustrates the stimuli used. The four columns correspond to the four experimental conditions.

Figure 2 shows the typical waveforms of the deepSC and the IC, as well as the schematic diagram of the stimuli. To make the images clearer, we applied band-pass filtering from 100 to 1000 Hz (different filtering frequencies did not change the significance of subsequent results). It can be observed that in the deepSC, the amplitude of the startle response was significantly inhibited after the presentation of prepulse stimuli. To test this inhibitory effect, we calculated the peak-to-peak value for each rat under the corresponding conditions, i.e., the maximum peak value minus the minimum peak value in the graph. Based on the peak-to-peak values, we calculated PPI and gap-PPI, with the calculation method being the response to startle stimulus alone (or startle stimulus under background noise condition) minus the response under the prepulse condition (or gap condition), divided by the response to startle stimulus alone (or startle stimulus under background noise condition). The calculation formulas for PPI and gap-PPI are as follows:


$$P\,P\,I=\frac{P\,u\,l\,s\,e\,\text{\_}\,o\,n\,l\,y\,\text{\_}\,v\,a\,l\,u\,e-P\,r\,e\,p\,u\,l\,s\,e\,\text{\_}\,p\,u\,l\,s\,e\,\text{\_}\,v\,a\,l\,u\,e}{P\,u\,l\,s\,e\,\text{\_}\,o\,n\,l\,y\,\text{\_}\,v\,a\,l\,u\,e}$$

$$g\,a\,p\,\text{\_}\,P\,P\,I=\frac{P\,u\,l\,s\,e\,\text{\_}\,b\,a\,c\,k\,g\,r\,o\,u\,n\,d\,\text{\_}\,v\,a\,l\,u\,e-g\,a\,p\,\text{\_}\,p\,u\,l\,s\,e\,\text{\_}\,v\,a\,l\,u\,e}{P\,u\,l\,s\,e\,\text{\_}\,b\,a\,c\,k\,g\,r\,o\,u\,n\,d\,\text{\_}\,v\,a\,l\,u\,e}$$

The results showed that the mean PPI induced by prepulse stimulation in the deepSC was 0.142, which was significantly different from zero, *t*(18) = 3.215, *p* = 0.005. The mean gap-PPI in the deepSC was −0.025, the mean PPI induced by prepulse stimulation in the IC was −0.082, and the mean gap-PPI in the IC was 0.001. Except for the PPI induced by prepulse stimulation in the deepSC, all other PPI or gap-PPI were not significantly different from zero (all *p* > 0.05).

### 3.3 Effects of narrowband noise as prepulse stimulus on the envelope and fine structure responses to startle stimulus

**FIGURE 3:**
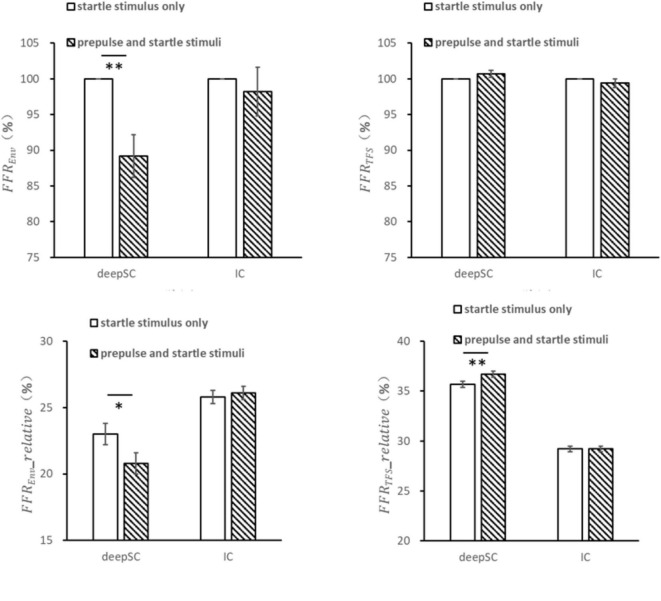
The effects of narrowband noise as a prepulse stimulus on the startle response in the deepSC and IC. The top two graphs represent the amplitude ratio of the envelope and fine structure components, using the startle response without the prepulse stimulus as the baseline. The bottom two graphs represent the relative amplitude of the envelope and fine structure components. *p < 0.05, **p < 0.01. Error bars: standard error.

For the condition where narrowband noise was used as the prepulse stimulus, the envelope response of the startle stimulus in the deepSC and IC was observed (Figure 3). Due to the variability in neural responses across different electrodes for each rat, the response to the startle stimulus alone was normalized to 1, and the response to the startle stimulus in the presence of the prepulse stimulus was expressed as a ratio relative to the startle-only response. In the deepSC, the envelope response to the startle stimulus was significantly inhibited by the prepulse stimulus, with the absolute value of the envelope response in the presence of the prepulse stimulus being significantly lower than the response to the startle stimulus alone, *F*(1,18) = 13.148, *p* = 0.002, η_*p*_^2^ = 0.422. In contrast, the envelope response to the startle stimulus in the IC was not significantly affected by the prepulse stimulus, *F*(1,18) = 0.280, *p* = 0.603, η_*p*_^2^ = 0.015. For the fine structure component, there were no significant differences in the startle response in both the deepSC and IC, regardless of the presence of the prepulse stimulus.

The relative amplitude of the envelope component within the frequency range was obtained by dividing the amplitude within the envelope frequency range by the total amplitude within 5000 Hz. The relative envelope response to the startle stimulus in the deepSC was significantly inhibited by the prepulse stimulus, *F*(1,18) = 8.135, *p* = 0.011, η_*p*_^2^ = 0.311, while the relative envelope response in the IC was not significantly affected by the prepulse stimulus.

The relative amplitude of the fine structure component within the frequency range was obtained by dividing the amplitude within the fine structure frequency range by the total amplitude within 5000 Hz. The relative fine structure response to the startle stimulus in the deepSC was significantly influenced by the prepulse stimulus, with the relative fine structure value being significantly higher in the presence of the prepulse stimulus compared to the startle-only condition, *F*(1,18) = 9.074, *p* = 0.007, η_*p*_^2^ = 0.335. This increase could be due to the relative decrease in the envelope component amplitude. In contrast, the relative fine structure response to the startle stimulus in the IC was not significantly influenced by the prepulse stimulus.

### 3.4 Effects of gap as prepulse stimulus on the envelope and fine structure responses to startle stimulus

**FIGURE 4:**
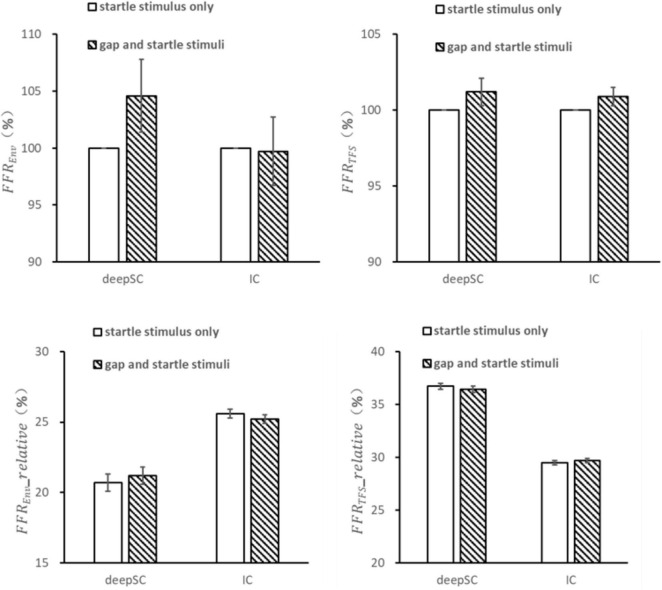
The effects of a gap as a prepulse stimulus on the startle response in the deepSC and IC. The top two graphs represent the amplitude ratio of the envelope and fine structure components, using the startle response without the gap as the baseline. The bottom two graphs represent the relative amplitude of the envelope and fine structure components. Error bars: standard error.

For the condition where an energy gap embedded in continuous narrowband noise was used as the prepulse stimulus, the responses to the startle stimulus in the deepSC and IC were observed (Figure 4). Unlike the condition with narrowband noise as the prepulse stimulus, there were no significant differences in the envelope and fine structure responses to the startle stimulus in both the deepSC and IC when the energy gap was used as the prepulse stimulus (all *p* > 0.05). Similarly, there were no significant differences in the relative amplitude of the envelope and fine structure components within the 5000 Hz frequency range.

## 4 Discussion

### 4.1 Inhibitory effect of prepulse stimuli on the envelope response to startle stimuli

Previous research on the attentional modulation of PPI has mostly focused on the Prepulse stimulus (Du et al., 2009a,2009b,^2010^), but the inhibition of the startle reflex is the outcome of attentional modulation of PPI. Therefore, studying the changes in the neural representation of the startle stimulus is also crucial. In human experiments, it has been suggested that attentional modulation of PPI requires at least 100 milliseconds. Therefore, by examining the neural responses evoked by startle stimuli at 60-millisecond and 120-millisecond intervals, it is possible to identify the brain regions specific to attentional modulation of PPI (Heidinger et al., 2019). Numerous animal experiments have found that a 50-millisecond interval between the Prepulse stimulus and the startle stimulus can also effectively introduce attentional modulation (Lei et al., 2014; Wu et al., 2016; Ding et al., 2019; Meng et al., 2020).

In this study, electrophysiological recordings were conducted in the deepSC and IC. Frequency following responses (FFRs) are neural discharges from populations of neurons that can reflect the processing characteristics of various sound stimuli, such as pure tones, narrowband noise, speech, and music (Skoe and Kraus, 2010). Our results showed that in the deepSC, the Prepulse stimulus significantly inhibited the response to the subsequent startle stimulus. Specifically, the envelope component of the startle stimulus response in the deepSC was significantly inhibited by the narrowband noise Prepulse stimulus, while the fine structure was not affected. Due to the decreased expression of the envelope component, the relative amplitude of the fine structure component increased. This inhibitory effect of the Prepulse stimulus on the startle stimulus was only observed in the deepSC and not in the IC or when the Prepulse stimulus was a gap.

The deepSC receives multimodal sensory inputs, which likely contributes to its integral function in the PPI circuit. The inhibition of the envelope component of the startle response by narrowband noise prepulse stimuli, observed exclusively in the deepSC and not in the IC or with gap prepulse stimuli, highlights this region’s specific role in auditory PPI. This specificity aligns with previous research indicating that the deepSC integrates signals from various sensory channels to regulate the startle reflex (Zou et al., 2007; Li et al., 2009). Moreover, the differential effects of narrowband noise and gap prepulse stimuli suggest distinct neural pathways for sound-induced PPI and gap-PPI, supporting the notion of multiple branches in the PPI circuit, as reviewed by Lei et al. (2021). These insights into the neural mechanisms underlying different forms of PPI contribute to a deeper understanding of sensory processing and may inform therapeutic strategies for disorders involving impaired PPI, such as tinnitus.

Interestingly, a study based on rhesus monkeys and our study together provide cross-species evidence. Waguespack et al. (2020) found that inhibition of the deep and intermediate layers of the superior colliculus significantly disrupted PPI in rhesus monkeys, while our study also demonstrated that the deepSC play a crucial role in PPI in rats. These results consistently support the importance of the superior colliculus in the PPI circuit, indicating that the superior colliculus is essential for normal PPI function in both primates and rodents. However, the species-specific differences should not be overlooked. Waguespack et al. (2020) study pointed out that while the role of the superior colliculus in PPI shows similarities across species, the neural circuits regulating PPI differ significantly between primates and rodents. For example, the study found that inhibition of the substantia nigra pars reticulata (SNpr) has opposite effects on PPI in these species. This highlights the importance of considering species-specific neural circuit differences in cross-species research. Furthermore, Waguespack et al. (2020) study provides an important reference point for our research, particularly regarding the complexity and diversity of PPI circuits. Our results showed that narrowband noise as a prepulse stimulus significantly inhibited the envelope component of the startle response in the deepSC, an effect not observed in the inferior colliculus or with gap prepulse stimuli. Waguespack et al. (2020) findings further support the idea that the PPI circuit may have multiple branches, with some bypassing the superior colliculus and others passing through it. This multi-branch neural circuit helps explain the mechanisms of PPI regulation and highlights the specific roles of different brain regions in PPI.

### 4.2 Different neural bases of gap-PPI and narrowband noise as prepulse stimuli

This study found that when narrowband noise was used as the Prepulse stimulus, the response strength to the subsequent startle stimulus in the deepSC decreased, as reflected in the absolute and relative amplitude of the envelope component. However, the response amplitude of the startle stimulus was not affected by a gap embedded in background noise. Previous research has shown that Gap-PPI and sound-induced PPI have different neural circuits. Our results also suggest that the neural basis of Gap-PPI may differ from that of sound-induced PPI. Some studies have used c-Fos labeling to investigate the neural circuits of PPI (Takahashi et al., 2007; Arai et al., 2008). In a study on the LGP using this method, it was found that the LGP is involved in sound-induced PPI but not in Gap-PPI (Moreno-Paublete et al., 2017). Previous studies have shown that reversible inhibition of the auditory cortex by high concentrations of potassium chloride disrupts Gap-PPI but not sound-induced PPI (Ison et al., 1991). This indicates that compared to regular PPI, Gap-PPI depends on higher brain regions such as the auditory cortex. c-Fos labeling studies have also confirmed this, showing that Gap-PPI triggers c-Fos activation in the auditory cortex (Moreno-Paublete et al., 2017). In our experiment, the rats were deeply anesthetized during electrophysiological recording, which may have partially inhibited the function of the auditory cortex.

As mentioned earlier, Gap-PPI is commonly used in tinnitus research, and studies have shown that many neurons in the auditory cortexare associated with tinnitus (Elgoyhen et al., 2015). Additionally, changes in neural activity have been observed in the ventral cochlear nucleus, dorsal cochlear nucleus, and medial geniculate body (Kalappa et al., 2014), among other areas. Reviews by Shore and Wu (2019) and Hockley and Shore (2023) provide comprehensive insights into these changes. Interestingly, data on whether there are neural changes in the IC associated with tinnitus is very mixed. In this study, we also observed a lack of gap effect, which provides new data on the relationship between the IC and tinnitus. However, the IC is a complex brain region, and further detailed research is needed to elucidate the relationship between the IC, tinnitus, and gap-PPI.

It is possible that abnormal function of the auditory cortex in tinnitus patients prevents effective detection of gaps, rendering them unable to recognize the prepulse stimulus in Gap-PPI and consequently unable to produce effective startle reflex inhibition. The difference between the circuits of regular PPI and Gap-PPI may lie at the forebrain level. It remains unclear whether regular PPI and Gap-PPI share the same basic circuitry at the lower levels. We speculate that the Gap-PPI circuit is more complex than that of regular PPI because gap detection is more intricate than detecting ordinary sound stimuli. The role of the auditory cortex may be critical in gap detection rather than in the formation of prepulse inhibition. Failure to detect the stimulus naturally results in the absence of a gating mechanism and subsequent inhibition. Based on this, we hypothesize that there is considerable similarity between the lower-level circuits of Gap-PPI and regular PPI. Although our experiment observed different electrophysiological responses to the startle stimulus in the deep superior colliculus (deepSC) when using the two types of prepulse stimuli, this difference may be due to the inability of anesthetized animals to effectively detect the gap embedded in background noise or to perceive the gap as a complete auditory object or event.

According to the processing-protection theory (Graham, 1975), the brain has the ability to protect ongoing cognitive activities by activating a gating mechanism to prevent interference from subsequent startle stimuli. This is known as Prepulse inhibition of the startle reflex. However, if the Prepulse stimulus is not processed, the gating mechanism and subsequent inhibition will not occur. Thus, successful detection is crucial for Gap-PPI. In our experiment, it may be that the gap is not processed in the deepSC, and therefore, the expression of the subsequent startle stimulus is not affected.

### 4.3 Limitations

This study has several limitations that should be considered. First, the use of deep anesthesia may affect the function of higher auditory brain regions, such as the auditory cortex, potentially influencing the detection of gaps and subsequent Gap-PPI. Second, although FFR is a valuable technique for studying auditory processing, it has some limitations. FFR provides limited spatial resolution, capturing overall brain activity but not precisely localizing neural sites. The recorded signals can be complex in origin and influenced by background noise and electrode placement, making interpretation of the results challenging. Additionally, there are significant individual differences in FFR due to variations in hearing abilities, making cross-subject comparisons challenging. Third, although the sample size of 19 rats is adequate for statistical analysis, it may limit the generalizability of the findings. Furthermore, only male rats were used in our study, which means the study cannot address potential gender differences. Finally, the experimental conditions, including the specific types and parameters of prepulse and startle stimuli used, may not fully replicate conditions in natural settings, potentially limiting the applicability of the results to real-world scenarios. Future studies should address these limitations by using more precise recording techniques and a broader range of stimulus conditions to provide a more comprehensive understanding of PPI mechanisms.

## 5 Summary

Through electrophysiological recordings of the startle stimulus in the deepSC, we found that the envelope expression of the startle stimulus in the deepSC was significantly inhibited by the Prepulse stimulus. When the Prepulse stimulus was present, both the absolute and relative amplitudes of the envelope response in the deepSC were significantly reduced, while the IC, used as a control brain region, did not exhibit this inhibitory effect.

## Data Availability

The raw data supporting the conclusions of this article will be made available by the authors, without undue reservation.
